# Synthesis, Stability, and Crystal Structure of CaTi_2_O_4_‑Type ZnCr_2_O_4_: High-Pressure Crystal Chemistry of Postspinel Phases

**DOI:** 10.1021/acs.inorgchem.5c01473

**Published:** 2025-06-26

**Authors:** Takayuki Ishii, Yudai Masaki, Alan B. Woodland, Yoshiyuki Inaguma, Hiroshi Kojitani, Masaki Akaogi

**Affiliations:** † Institute for Planetary Materials, 12997 Okayama University, Misasa, 682-0193 Tottori, Japan; ‡ Department of Chemistry, Gakushuin University, Mejiro, Toshima-ku, Tokyo 171-8588, Japan; § Institut für Geowissenschaften, Goethe-Universität Frankfurt, Altenhöferallee 1, 60438 Frankfurt am Main, Germany; ∥ Geochemical Research Center, Graduate School of Science, The University of Tokyo, Hongo, Bunkyo-ku, Tokyo 113-0033, Japan

## Abstract

High-pressure phases of spinel-type *A* ^*2+*^ *B*_2_^*3+*^O_4_, called postspinel phases, have been of great interest in materials science for the appearance of novel physical properties and geoscience for understanding the Earth’s deep interior. The CaFe_2_O_4_- and CaTi_2_O_4_-structured phases are representative postspinel phases. To design a postspinel phase, it is useful to construct a prediction diagram using crystallographic parameters such as ionic radius. Especially, the appearance of the CaTi_2_O_4_-type phase is rare in high-pressure–temperature space, and therefore, its further exploration is desired to develop its material group. Our previous study constructed a structural prediction diagram of *A* ^*2+*^ *B*_2_^*3+*^O_4_ postspinel phases based on ionic radii. Based on the prediction diagram, we here investigated the system ZnCr_2_O_4_ at high-pressure–temperature conditions to explore the stability field of the CaTi_2_O_4_-type phase. We found that the spinel-type phase transformed to the CaTi_2_O_4_-structured phase at 23–24 GPa and 1000–1400 °C. Rietveld structure analysis of the recovered high-pressure phase yielded lattice parameters for the CaTi_2_O_4_-type phase of *a* = 2.8634(1) Å, *b* = 9.4990(4) Å, *c* = 9.7568(3) Å, and *Z* = 4 (space group: *Cmcm*). Thus, our prediction diagram with ionic radii successfully predicted the structure types of an *A* ^*2+*^ *B*_2_^*3+*^O_4_ postspinel phase and is therefore useful to design a novel postspinel phase.

## Graphical abstract

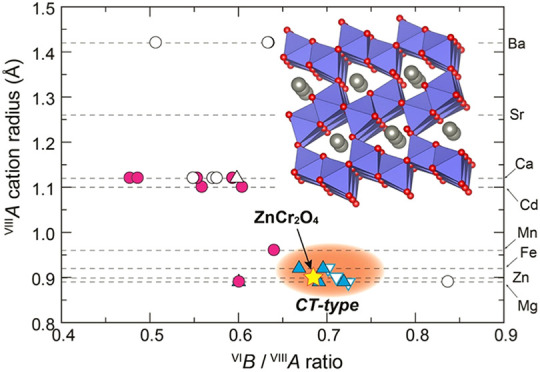

## Introduction

Postspinel phases with the *A* ^2+^ *B*_2_^3+^O_4_ composition such as calcium ferrite (CF)-, calcium titanite (CT)-, and calcium manganate (CM)-type phases,^1^ defined as high-pressure polymorphs of a spinel-type phase, have attracted interest in the materials sciences and geoscience because of their distinctive structures as described below. The CF-, CT-, and CM-type structure types belong to space groups *Pnma* (no. 62), *Cmcm* (no. 63), and *Pbcm* (no. 57), respectively. These structures consist of double chains of edge-sharing *B*O_6_ octahedra running parallel to one of the orthorhombic cell axes. The four double chains linked with one of their corners form a tunnel-like space, in which *A* ^2+^ cations are accommodated. The CF- and CT/CM-type structures can be distinguished by characteristics of the *B*O_6_-octahedral frameworks,^2,3^ noting that the CM-type phase appears when the *B* cation is a Jahn–Teller active cation such as Mn^3+^.

In materials science, CF-type phases have been intensively synthesized as expected novel magnetic and cathode materials because of their specific structure. A pseudo-one-dimensional structure of double chains with magnetic and/or mixed-valence cations forms a magnetically frustrated spin system and/or a highly anisotropic electrical path,^4–7^ respectively. In addition, a pseudo-one-dimensional structure of *A*-site cations in the octahedral tunnel space can make highly ionic conducting materials due to the large thermal vibration of *A* cations in the tunnel structure.^8,9^ From the viewpoint of geoscience, CF- and CT-type postspinel phases can form in chemically distinctive crustal materials compared with the average composition of the Earth’s mantle^10–12^ when they are subducted to depths of > ∼700 km.^13–17^ These phases can host relatively large incompatible cations such as Na^+^, because of the relatively large tunnel-shaped space surrounded by octahedra.^18^ Thus, these phases play an important role in the material cycle in the Earth’s interior. In addition, the physical properties of the CF-type phase provide hints to understand the structure and evolution of the Earth’s interior.^19,20^ In particular, the postspinel transitions in MgAl_2_O_4_, which is a major component of Al-rich phases in the deep mantle, have been paid special attention. MgAl_2_O_4_ spinel transforms to CF and CT structure types at ∼25 and ∼40 GPa, respectively.^21–23^ However, because the transition pressures are relatively high, phase stabilities and physical properties of the postspinel phases of MgAl_2_O_4_ are not fully understood, especially those of the CT-type structure. For this reason, postspinel phases in different chemical systems can be good analogues to understand postspinel phases in the Earth’s interior.

In both of the fields, high-pressure–temperature phase relations of postspinel phases with *A* ^2+^ *B*_2_^3+^O_4_ compositions are intensively investigated in simple systems.^22,24–29^ High-pressure experimental studies indicate that most spinel-type *AB*_2_O_4_ phases first decompose into two different types of phases: rock-salt-type *A*O + corundum (Cor)-type *B*_2_O_3_, modified ludwigite (mLd)-type *A*_2_ *B*_2_O_5_ + Cor-type *B*_2_O_3_, or CaFe_3_O_5_-type *A*_2_ *B*_2_O_5_ + Cor-type *B*_2_O_3_. At higher pressure, these assemblages recombine into a single postspinel phase of *AB*_2_O_4_ with CF- and/or CT/CM-type structures.^3^ Note that mLd- and CaFe_3_O_5_-type structures also have octahedral frameworks with tunnel-like spaces that accommodate relatively large cations on their *A*-site.^30,31^

Compared with CF-type compounds, limited CT-type high-pressure compounds have been reported.^22,24,26,27,32,33^ Our previous study attempted to categorize structure types of *AB*_2_O_4_ postspinel phases using ionic radii^2^ as shown in Figure 1. This diagram shows that CT-type phase appears in limited cation variations compared with the CF-type phase. This is probably because of the flexibility of the octahedral tunnel space: the tunnel space of the CT-type structure with a higher symmetry is more rigid than that of the CF-type one, resulting in the accommodation of cations only with selected sizes (∼0.9 Å for ^VIII^ *A* ^2+^ and 0.6–0.65 Å for ^VI^ *B* ^3+^).

**Figure 1:**
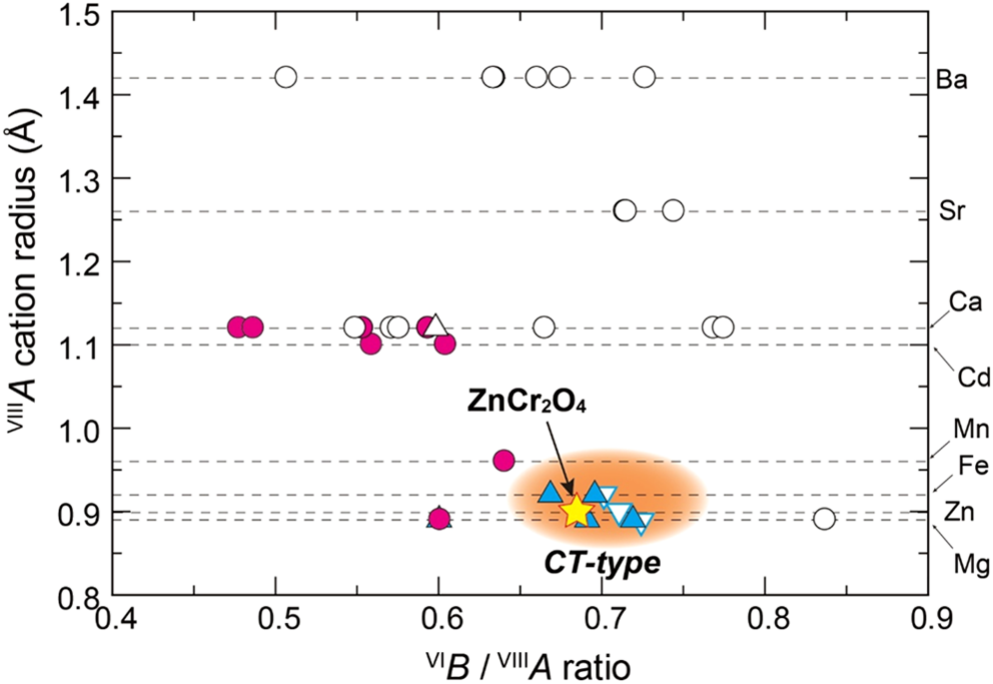
Structural prediction diagram with ionic radii of ^VIII^ *A* ^2+^ and ^VI^ *B* ^3+^/^VIII^ *A* ^2+^ radius ratios. Closed triangles and closed circles indicate that CaTi_2_O_4_ (CT)-type and CaFe_2_O_4_ (CF)-type phases were synthesized, respectively, at high-pressure–temperature conditions and confirmed to be quenchable (ref 2 and references therein). Open circles and triangles indicate that CF-type and CT-type phases were synthesized at high temperatures, respectively (ref 2 and references therein). Open inversed triangles indicate CT-type phases identified under high pressures.^24,33^ The star symbol is located at the ZnCr_2_O_4_. The orange region denotes where a CT-type phase is expected to appear. Reproduced and modified from ref 2. Copyright 2018 American Chemical Society.

In this study, based on this diagram, we further attempted to explore a new CT-type compound. We selected Zn^2+^ (0.90 Å for an 8-fold coordination) and Cr^3+^ (0.615 Å for a 6-fold coordination) as *A*- and *B*-site cations, respectively, which are suitable to synthesize a CT-type compound according to Figure 1. Spinel-type ZnCr_2_O_4_ is of great interest as a technological material due to its magnetic, electronic, and catalytic properties.^35–37^ It is reported that this phase experiences high-pressure transitions, in which the decomposition of the spinel phase to ZnO + Cr_2_O_3_ and/or orthorhombic phases appears at high pressures.^38–40^ However, its high-pressure–temperature phase relations are poorly constrained.^39,40^ Therefore, we investigated the high-pressure–temperature phase relations in the system ZnCr_2_O_4_. We will report a newly found CT-type phase and its refined crystal structure, allowing further discussion of the crystallographic categorization of the postspinel group.

## Experimental Details

### Starting Materials

High-pressure–high-temperature experiments were conducted using a presynthesized spinel-type ZnCr_2_O_4_. The starting material was prepared from a mixture of reagent-grade ZnO (Alfa Aesar, 99.99%) and Cr_2_O_3_ (Kanto Kagaku Co., >99%) in a molar ratio of 1:1 at 1 bar and 925 °C for 15 h. Powder X-ray diffraction (XRD) confirmed the starting material to be single phase with a lattice parameter of *a* = 8.3266(9) Å, which is consistent with that reported by ref 41. We note that the chemical compositions of the spinel-type phase were determined to be Zn/Cr = 1.03(3):1.98(2) in atomic ratio on a four-oxygen basis, and no other elements were detected. In addition, synchrotron X-ray diffraction study suggested no secondary phase except for Pt from the capsule material as shown later, implying that any impurities in the starting chemicals had not influenced the phase relations in the ZnCr_2_O_4_ system and the crystal structure of the recovered high-pressure phase.

### High-Pressure–High-Temperature Experiments

Phase relation experiments were performed at 20–24 GPa and 800–1400 °C for 60–180 min using the Kawai-type multianvil high-pressure apparatus installed at Gakushuin University, Japan, which employs tungsten carbide anvils with a 2.5 mm truncated edge length. A cylindrical Pt heater was inserted into the center of a 5 wt % Cr_2_O_3_-doped MgO octahedral pressure medium with a 7 mm edge length. The powdered starting material was placed inside the heater and closed off at each end with a Pt disk, followed by a LaCrO_3_ lid. The heater/capsule was surrounded by a LaCrO_3_ sleeve. Sample temperatures were monitored using a Pt/Pt-13%Rh thermocouple at the center part of the heater surface with no correction for the pressure effect on the electromotive force of the thermocouple. The sample for structure analysis was synthesized using the Kawai-type multianvil apparatus with the Osugi-type guide block (IRIS-15)^42,43^ installed at Bayerisches Geoinstitut, University of Bayreuth. The 7 mm pressure medium was used by combining with tungsten carbide anvils with a 3 mm truncation. A LaCrO_3_ heater was located at the center of the pressure medium. A Pt-foil capsule was used to pack the starting sample, which was put in a MgO capsule inside the heater. A W_97_Re_3_–W_75_Re_25_ thermocouple was used to monitor temperatures. Pressure calibration was conducted against press load^44^ using forsterite-wadsleyite^45^ and wadsleyite-ringwoodite^46^ transitions in Mg_2_SiO_4_, akimotoite-bridgmanite transition in MgSiO_3_,^47,48^ and pyrope–[bridgmanite+corundum] transition in Mg_3_Al_2_Si_3_O_12_ ^47,49^ at 1600 °C. The cell assembly was compressed to a target press load for 3–4 h at room temperature and then heated to the desired temperature at a rate of ∼100 °C/min. The sample was quenched by turning off the electrical power after the target temperature was kept for a certain duration (Table 1). The sample pressure was slowly released to ambient conditions overnight.

**Table 1:** Results of High-Pressure, High-Temperature Experiments^a^

run no.	pressure (GPa)	temperature (°C)	time (min)	phases
180828	20	800	120	Sp
180928	23	800	180	Sp
180904	20	1000	60	Sp
181130	23	1000	180	Sp
181107	24	1000	90	CT+Sp^c^
180922	23	1200	90	Sp
181031	24	1200	90	CT+Sp^c^
181115	25	1200	120	CT+Sp^c^
181023	26	1200	90	^b^CT
181210	22	1400	90	Sp
181010	23	1400	90	CT+Sp^c^
181112	24	1400	90	CT+Sp^c^

a Abbreviations: Sp, spinel-type phase; CT, CaTi_2_O_4_-type phase.

b The recovered sample was used for synchrotron X-ray diffraction measurements to conduct the Rietveld refinements.

c Presence of spinel-type phase was interpreted to be metastable (see the main text).

### Analyses of Recovered Samples

The starting material and recovered samples were identified using a powder X-ray diffractometer (Rigaku RINT 2500 V) with monochromatized Cr Kα radiation operated at 45 kV and 250 mA. Lattice parameters of these samples were also determined from the XRD patterns using DICVOL06 software.^50^ Compositions of the recovered samples were checked using a field-emission-type electron probe microanalyzer (EPMA) (JEOL JXA8800) with operating conditions of 15 kV and 5 nA. Synthetic ZnO and Cr_2_O_3_ eskolaite were used as standards.

The XRD data for the Rietveld analysis of a recovered phase was collected by angle-dispersive synchrotron XRD in the BL02B2 at SPring-8. The data collection at room temperature was conducted using a Debye–Scherrer camera with an imaging plate in a 2θ range of 0–78° with a 0.006° step while rotating the sample. The wavelength (λ = 0.41990 Å) of incident X-rays was calibrated with fluorite-type CeO_2_ using the same imaging plate. The polycrystalline sample synthesized at 26 GPa and 1200 °C was ground in an agate mortar and then was packed in a Lindemann glass capillary.

The Rietveld analysis of the diffraction data was performed with the RIETAN-FP/VENUS package.^51^ The structure of CT-type MgCr_2_O_4_ was adopted as the initial structure model of the ZnCr_2_O_4_ sample. The lattice parameters for the Rietveld refinement were fixed to the values determined with the laboratory powder XRD pattern (Cr Kα) described above. Pt metal was included in the Rietveld analysis as a minor impurity. A background of the XRD pattern was fit by using a Legendre polynomial function constructed with 12 parameters. Finally, we simultaneously refined scale factors of phases included, atomic coordinates, and isotropic atomic displacement parameters of each atom, using a split-type pseudo-Voigt profile fitting function.^52^

## Results and Discussion

### Phase Relations

We conducted 12 experiments to constrain the phase relations (Table 1 and Figures 2 and 3). XRD profiles of recovered samples showed diffraction peaks different from those of the spinel-type phase above 24–26 GPa at 1000–1400 °C (Figure 2). These peaks were not from decomposition products such as ZnO and Cr_2_O_3_, suggesting the formation of a new ZnCr_2_O_4_ phase. The composition of the recovered sample was determined to have Zn/Cr = 1.02(2):1.99(2) in atomic ratio on a four-oxygen basis, suggesting that the new phase is stoichiometric. Spinel-type ZnCr_2_O_4_ directly thus transforms to a new phase at 23–24 GPa and 1000–1400 °C, showing a negative Clapeyron slope (Figure 3). The presence of residual peaks of the spinel-type phase was presumably due to sluggish kinetics of this transformation at the pressure–temperature conditions. It may also be attributed to the effect of a small temperature gradient in the sample space, particularly in the runs near the transition boundary. Another possible reason is a back-transformation from the new phase due to a slight pressure decrease during heating.^53^ The fact that spinel-type and new phases coexist further supports the absence of decomposition phases in this system. As shown below, the Rietveld analysis of the sample recovered from 26 GPa and 1200 °C indicated that the new phase has a CT-type structure. The direct transformation from the spinel-type phase to the CT-type phase is the first report to our knowledge. In other systems such as FeV_2_O_4_, MgV_2_O_4_, MgCr_2_O_4_, and FeCr_2_O_4_, CT phases appear through decomposition phases of rock-salt-type *A*O or modified ludwigite-type *A*_2_ *B*_2_O_5_ + corundum-type *B*_2_O_3_ with increasing pressure.^2,26,27^ For ZnCr_2_O_4_, the density increase associated with the spinel-CT transition is ∼8%, using the lattice parameters of the CT phase shown below.

**Figure 2:**
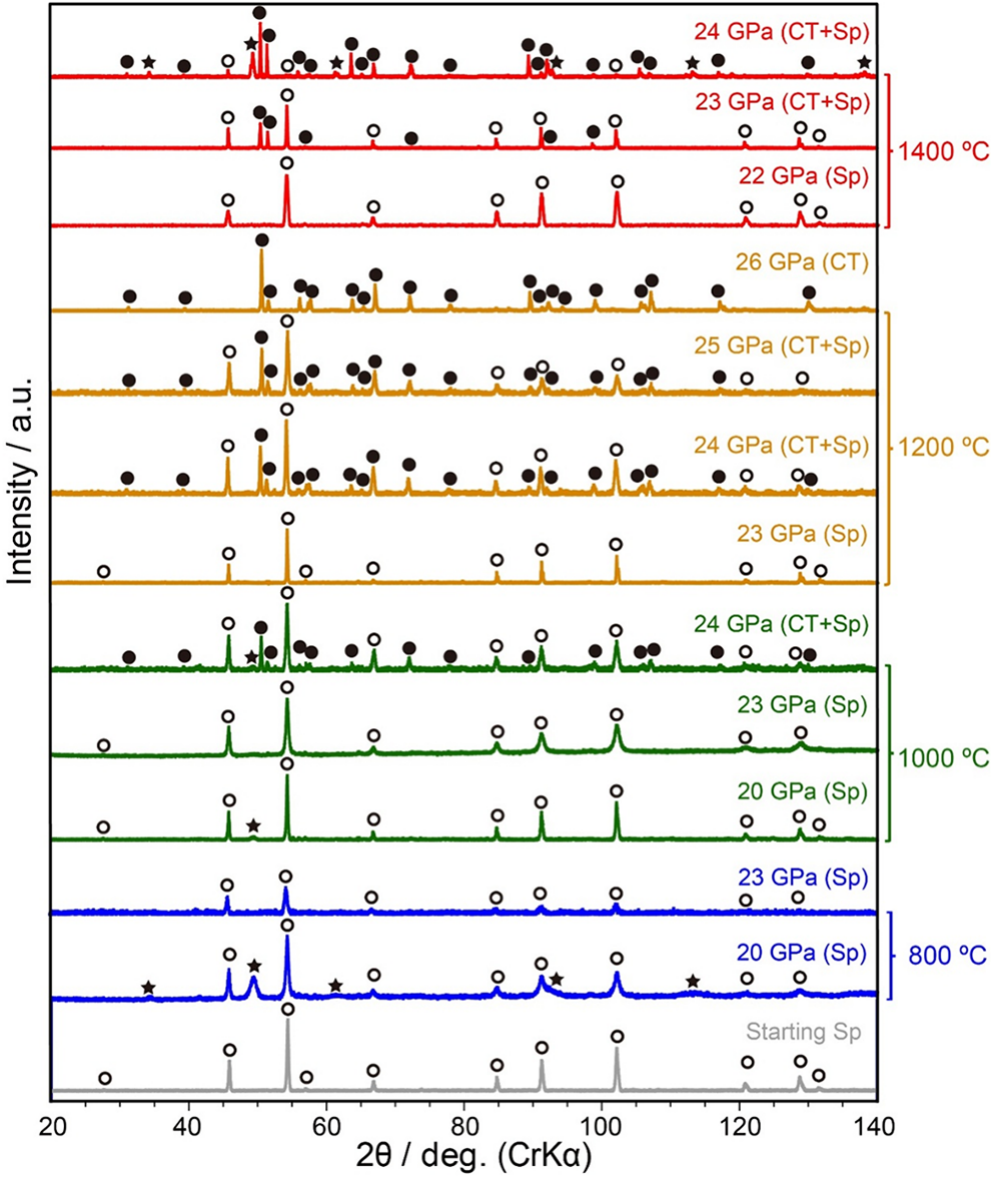
X-ray diffraction profiles of recovered samples under ambient conditions. Open circles: spinel-type phase (Sp); closed circles: CaTi_2_O_4_-type phase (CT); and closed stars: LaCrO_3_ thermal insulator.

**Figure 3:**
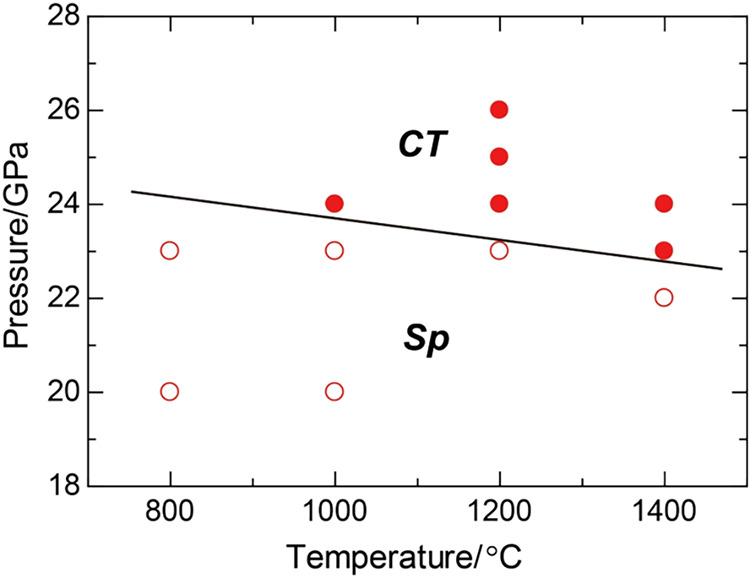
High-pressure–high-temperature phase relations in ZnCr_2_O_4_. Sp, spinel-type phase; CT, CaTi_2_O_4_-type phase. Open and closed circles express the runs at which Sp and CT, respectively, are stable. The solid line is the phase boundary between Sp and CT stability fields.

Spinel-type ZnCr_2_O_4_ has a completely normal spinel structure, in which Zn^2+^ is tetrahedrally coordinated and Cr^3+^ octahedrally coordinated with O^2–^.^54,55^ It is generally accepted that the stability of zinc oxide compounds with tetrahedrally coordinated Zn^2+^ at relatively low pressure is attributed to the strong tetrahedral preference of Zn^2+^ with the *d* ^10^ electron configuration. A typical example is ZnO, which transforms from the wurtzite type to the rock-salt type at ∼5 GPa.^56^ The coordination number of Zn^2+^ increases from 4 to 6 in the spinel-CT transition in ZnCr_2_O_4_, while that of Cr^3+^ does not change.

Here, we compare the transition in ZnCr_2_O_4_ with those in MgCr_2_O_4_ and FeCr_2_O_4_. Mg^2+^ (0.72 Å) and Fe^2+^ (0.78 Å) have similar ionic radii to Zn^2+^ (0.74 Å), where numbers in parentheses are 6-fold-coordinated cation radii.^34^ With increasing pressure, spinel-type MgCr_2_O_4_ and FeCr_2_O_4_ first decompose to modified ludwigite-type *A*_2_ *B*_2_O_5_ (or rock-salt-type *A*O) + corundum-type *B*_2_O_3_ at ∼12–15 GPa, which then combine into CF- (or CT-)­type phase at ∼16–20 GPa.^26,27^ In ZnCr_2_O_4_, however, our study shows that spinel-type ZnCr_2_O_4_ persists to 22–24 GPa and directly transforms to CT-type without decomposition reactions (Figures 2 and 3). The difference between the transition in ZnCr_2_O_4_ and those in MgCr_2_O_4_ and FeCr_2_O_4_ may be explained by the tetrahedral preference of Zn^2+^ at relatively low pressure,^57–59^ because *A* ^2+^ is in six coordination with O^2–^ in both the rock-salt-type (octahedral site) and mLd-type (octahedral and trigonal prism sites) structures.^31^ Spinel-type MnCr_2_O_4_ directly transforms to a CF-type phase at ∼10 GPa^2^. This may be derived from the fact that Mn^2+^ tends to prefer a tetrahedral coordination rather than the octahedral one,^57–59^ resulting in no transition to structures with an octahedral coordination. In addition, the reason for crystallizing to the CF-type structured phase may be due to a larger ionic radius of ^VI^Mn^2+^ (0.83 Å) than ^VI^Mg^2+^, ^VI^Fe^2+^, and ^VI^Zn^2+^.

A numerical study predicted that the decomposition of the spinel phase to ZnO + Cr_2_O_3_ should occur at 34 GPa and room temperature.^38^ The pressure condition is much higher than the spinel-CT-phase transition pressure (∼28 GPa) at room temperature estimated by extrapolating the present phase boundary, supporting the direct transformation to the CT-type phase. An experimental study investigated the transformation of spinel-type ZnCr_2_O_4_ up to 70 GPa at room temperature by Raman spectroscopy.^39^ They found that spinel-type ZnCr_2_O_4_ transforms to a CF- or CT-type phase at 17.5 GPa based on changes in Raman spectra. Another experimental study also synthesized an orthorhombic phase at 44 GPa by XRD.^40^ Our results clearly demonstrate that no CF-type phase is stable at least above 1000 °C and a negative slope for the transition from spinel to the CT-type phase is located around ∼23 GPa, suggesting that the high-pressure phase observed in the previous studies may be the CT-type ZnCr_2_O_4_. However, as mentioned above, the extrapolated transition pressure at room temperature is ∼28 GPa, which is not consistent with the transition pressure reported by ref 39. The reason for this discrepancy can be explained by their diamond anvil cell experiments without pressure medium, which may have produced nonhydrostatic condition, possibly making the lower transition pressure. Another possibility is that a CF-type phase or some unknown structured metastable phase was synthesized. Note that such a room-temperature transformation to the CT-type phase probably needs higher pressure than that of the equilibrium phase transition due to the necessity of a complicated rearrangement of cations from the spinel lattice.^60^

### Crystal Structure of the High-Pressure ZnCr_2_O_4_ Phase

Here, we describe the results of the structure analysis of the new ZnCr_2_O_4_ phase. The graphical result of the Rietveld refinement of this phase is shown in Figure 4. Tables 2 and 3 give the structural parameters and reliability indices (*R*_wp_, *R*_e_, *R*_B_, and *R*_F_) for the present analysis. The reliability indices converged to sufficiently small values (<7% for *R*_wp_ and <∼4% for *R*_B_ and *R*_F_), indicating that the recovered compound has the CT-type structure. The interatomic distances, angles, effective coordination numbers (*n*_c_),^61^ and bond valence sum values^62^ are provided in Table 3.

**Figure 4:**
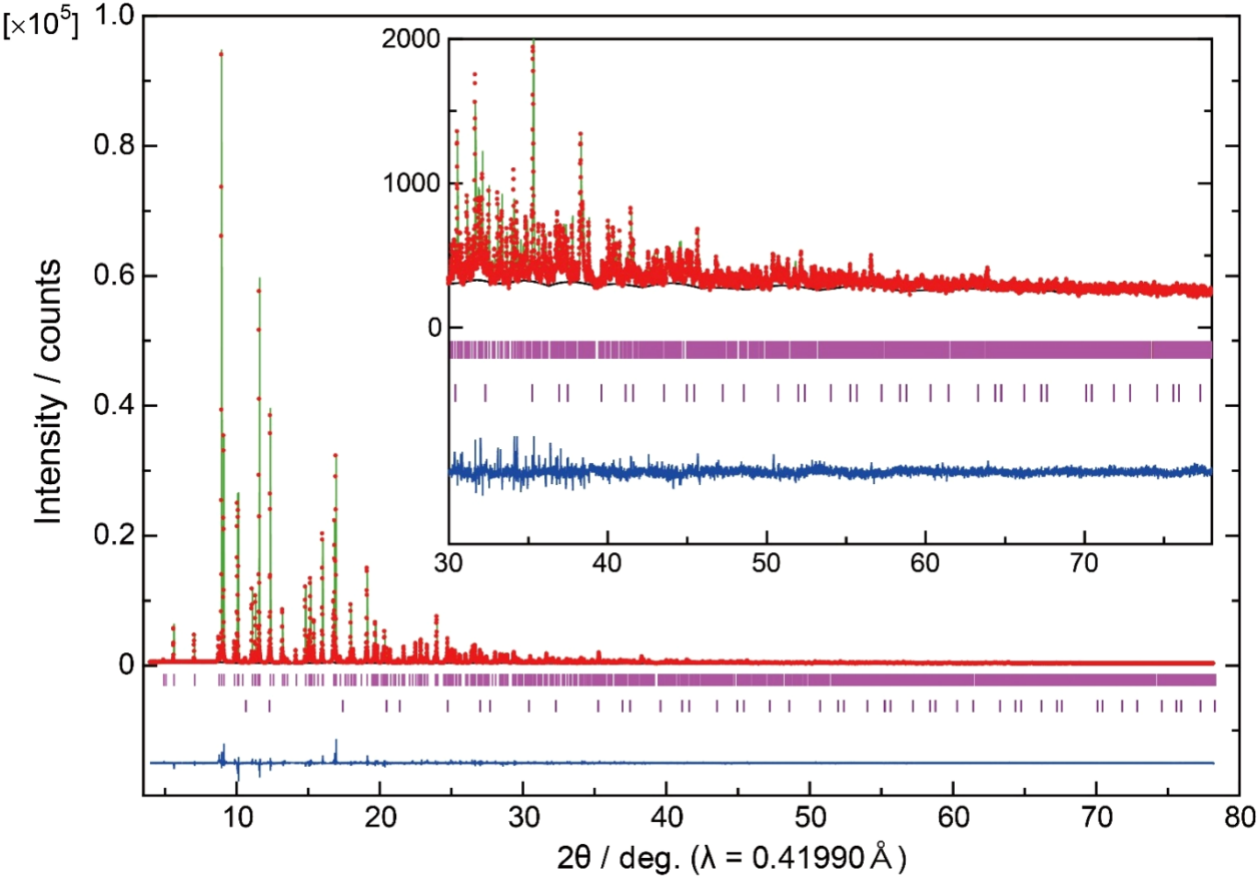
Results of the Rietveld refinements of CaTi_2_O_4_-type ZnCr_2_O_4_. The diffraction pattern was refined in the 2θ range of 4–78°. The inset is the result of the Rietveld refinements in the high-angle region (30–78°). The observed and calculated profiles are shown using dots (red) and solid lines (green), respectively. The residuals are shown at the bottom. The background profiles are shown by solid lines (black). Bragg peak positions are indicated by small ticks. The upper and lower ticks represent the Bragg peak positions of CT-type ZnCr_2_O_4_ and Pt, respectively.

**Table 2:** Atomic Coordinates and Isotropic Displacement Parameters for CaTi_2_O_4_-Type ZnCr_2_O4^b^ ^,^ ^c^

atom	Wyckoff site	*x*	*y*	*z*	^a^*U*_iso_ (Å^2^)
Zn	4*c*	0	0.11078(6)	1/4	0.0123(2)
Cr	*8f*	0	0.13166(7)	0.07088(5)	0.0047(1)
O1	4*a*	0	1/2	0	0.0090(6)
O2	4*c*	0	0.0396(3)	1/4	0.0049(5)
O3	*8f*	0	0.2319(2)	0.8902(2)	0.0084(4)

a Isotropic atomic displacement parameter.

b Refined lattice parameters of CaTi_2_O_4_-type ZnCr_2_O_4_: *a* = 2.8634(1) Å, *b* = 9.4990(4) Å, *c* = 9.7568(3) Å and *V* = 265.38(1) Å^3^ (*Z* = 4 and space group: *Cmcm*).

c *R*_wp_ = 6.965% and *R*_e_ = 4.036%. CaTi_2_O_4_-type ZnCr_2_O_4_: *R*_B_ = 3.744% and *R*_F_ = 4.319%. Pt: *R*_B_ = 4.584% and *R*_F_ = 3.712%.
$$\;R_{\mathrm{wp}}={\left\{\frac{\sum_{i}w_{i}{\left[y_{i}-f_{i}\left(x\right)\right]}^{2}}{\sum_{i}w_{i}y_{i}^{2}}\right\}}^{1/2},R_{B}=\left\{\frac{\sum_{K}\left|I_{0}\left(h_{K}\right)-I\left(h_{K}\right)\right|}{\sum_{K}I_{0}\left(h_{K}\right)}\right\},R_{F}=\left\{\frac{\sum_{K}\left|\left|F_{0}\left(h_{K}\right)\right|-\left|F\left(h_{K}\right)\right|\right|}{\sum_{K}\left|F_{0}\left(h_{K}\right)\right|}\right\},R_{e}={\left\{\frac{N-P}{\sum_{i}w_{i}y_{i}^{2}}\right\}}^{1/2}$$
where *y*_*i*_, *w*_*i*_, and *f*_*i*_(*x*) are the intensity observed at step *i*, the statistical weight, and theory intensity, respectively. *I*_0_(***h***_*K*_), *I*(***h***_*K*_), *F*_0_(***h***_*K*_), and *F*(***h***_*K*_) are the observed and calculated intensities and structure factors for reflection *K*, respectively. *N* and *P* are the number of all data points and refined parameters, respectively.

**Table 3:** Interatomic Distances and Angles in CaTi_2_O_4_-Type ZnCr_2_O_4_ ^a^

Bond Length (Å)			
Zn1–O1 × 2	2.6565(3)	Cr–O1 × 2	2.0229(5)
Zn1–O2 × 2	2.022(3)	Cr–O2	1.9542(14)
Zn1–O3 × 4	2.2901(16)	Cr–O3 × 2	1.9682(14)
^b^^VI^average	2.201	Cr–O3	2.004(3)
^b^^VIII^average	2.315	average	1.990
^b^ ^VI^ *n*_c_	4.92	*n*_*c*_	5.96
^b^ ^VIII^ *n*_c_	5.13	BVS	2.93
^b^^VI^BVS	1.67		
^b^^VIII^BVS	1.82		

Bond Angles (deg)					
O1–Cr–O1	90.10(3)	O2–Cr–O3	178.21(10)	O3–Cr–O3	81.77(8)
O1–Cr–O3	89.60(5)	Cr–O2–Cr	126.84(16)	Cr–O3–Cr	98.23(8)
Cr–O1–Cr	89.90(3)	O2–Zn–O2	90.13(12)	O3–Zn–O3	77.39(7)

a Abbreviations: *n*_c_: effective coordination number; BVS: bond valence sum value.

b VI and VIII indicate that these values were calculated using six and eight nearest neighbor oxygen-cation bonds, respectively.

The crystal structure of the CT-type ZnCr_2_O_4_ is shown in Figure 5. Edge-sharing CrO_6_ octahedra form a double-chain running parallel to the *a* axis. These double chains are linked by sharing corners, making tunnel-like spaces. Zn cations accommodated in the tunnel space occupy oxygen trigonal prism sites with two longer oxygen-cation distances. The *n*_c_ values of Zn^2+^ calculated from six and eight neighboring oxygen-Zn bonds were 4.92 and 5.13, respectively, which indicates that the two longest Zn–O bonds (Zn–O distance = 2.6565 Å) made a small contribution to the *n*_c_ value, and the oxygen coordination number of Zn can thus be considered to be practically six. The average Zn–O bond length in the ZnO_6_ prism was 2.201 Å. This value is consistent with the sum of the effective ionic radii (^VI^Zn^2+^ (0.74 Å) + ^VI^O^2–^ (1.40 Å) = 2.14 Å). The average Cr–O distance of the CrO_6_ octahedra was 2.004 Å, which is nearly the same as the sum of the effective ionic radii of ^VI^Cr^3+^ (0.615 Å) and ^VI^O^2–^, 2.015 Å. The bond valence sum values of Zn (+1.7) and Cr (+2.9) were also expected from the results of the chemical composition analysis. The refined structure had generally similar structural features (i.e., bond valence sum values, coordination environment, and interatomic angles) to those of CT-type MgCr_2_O_4_, FeCr_2_O_4_, MgV_2_O_4_, and FeV_2_O_4_. The angle formed by the corner-sharing octahedra (Cr–O2–Cr in Figure 5) has relatively high flexibility for changes in the tunnel size, because other parts are linked by edge-sharing. This angle increases with decreasing *B*-site cation radii: 123–124° for MgV_2_O_4_ and FeV_2_O_4_ (^VI^V = 0.64 Å), 124–127° for ZnCr_2_O_4_, MgCr_2_O_4_, and FeCr_2_O_4_ (^VI^Cr = 0.615 Å), and ∼129° for MgAl_2_O_4_ (^VI^Al = 0.535 Å) (Figure 6). This trend can be interpreted by the adjustment of tunnel size for *A*-site cations caused by changes in octahedral sizes.

**Figure 5:**
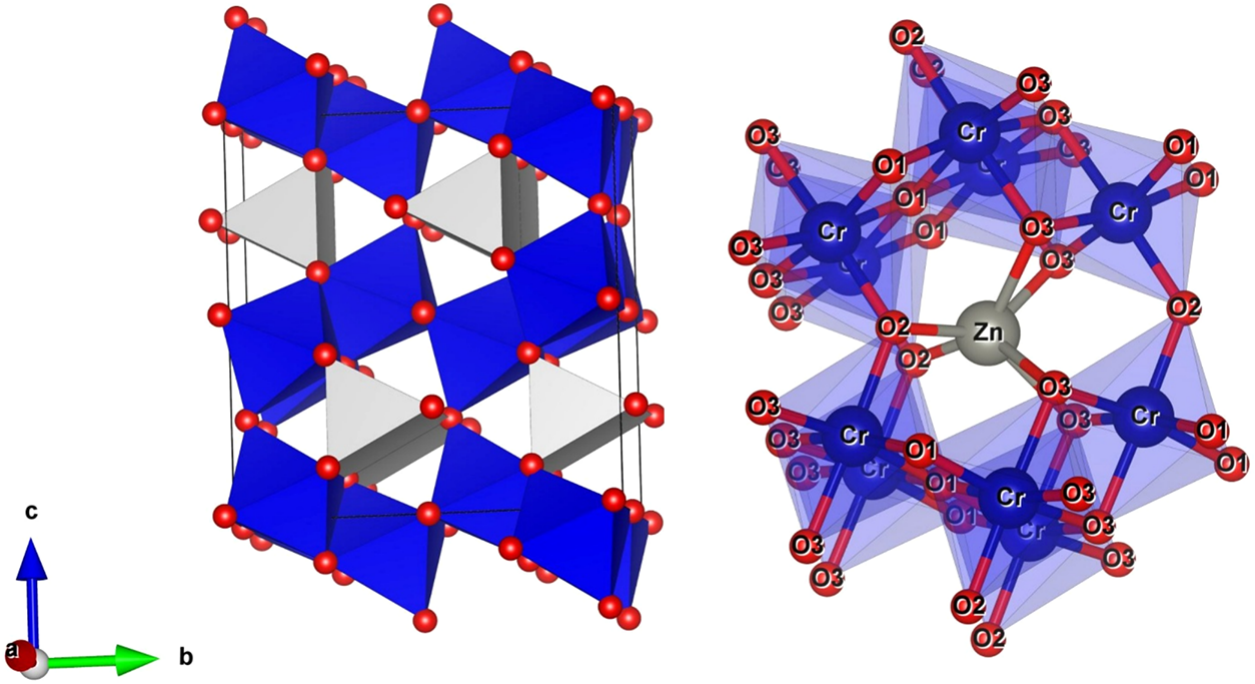
Refined structure of CT-type ZnCr_2_O_4_ viewed from the *a* axis. A solid box is a unit cell. Red spheres are oxygen. Blue octahedra are CrO_6_. Gray trigonal prism is ZnO_6_. The right figure shows detailed coordination environments of each site.

**Figure 6:**
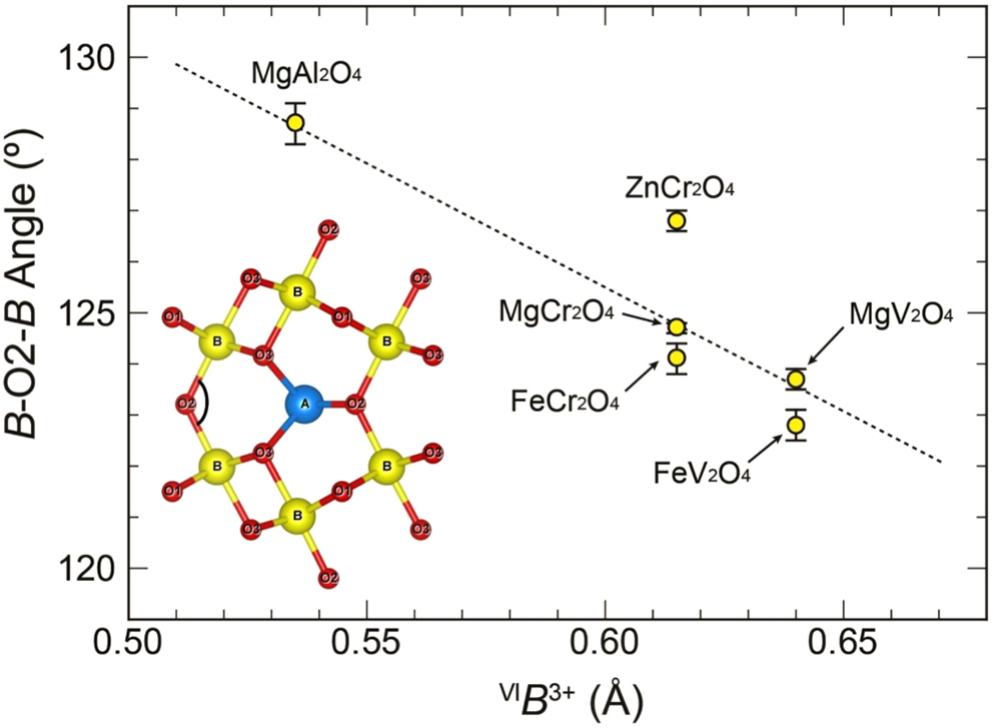
Relationship between the B-site cation size and angle formed by the corner-sharing octahedra at ambient conditions (Cr–O2–Cr in Figure 5) in CaTi_2_O_4_-type phases. The dashed line is drawn using data of Mg*B*_2_^3+^O_4_ phases (B = Al, Cr, V).

### Design of New CaTi_2_O_4_-Type Postspinel Phases and Future Perspectives

Our study discovered a new compound with a CT-type structure based on our structural prediction diagram. The prediction diagram showed a wide range of appearance for the CF-type phase,^2^ whereas the CT-type phase has a much more limited range. The present study demonstrates the usefulness of the diagram (Figure 1) to design the synthesis of new postspinel phases. We found that zinc compounds can be promising candidates to further synthesize a CT-type phase with transition-metal cations such as V^3+^ (0.64 Å) as discovered in MgV_2_O_4_ and FeV_2_O_4_ systems.

It is worthwhile mentioning that the CT-type structure is a high-pressure structure of CF type as demonstrated in previous studies.^1,23,26^ In addition, the CT-type phase is generally stable at higher temperatures than the CF-type phase, and the CF-CT-phase boundary generally has a great steep negative slope.^23,26^ Therefore, it is also possible that the CT-type phase can be synthesized at higher pressures and/or temperatures where the CF-type phase is stable. We note that multianvil technique is suitable to explore postspinel phases because of its large volume and homogeneous pressure–temperature field.^63^ We have explored postspinel phases at extreme conditions up to ∼30 GPa, which is almost the upper limit to apply conventional multianvil technology. The recent technical developments of multianvil experiments will facilitate their further exploration up to 65 GPa.^64–66^

Spinel-type ZnCr_2_O_4_ has a magnetically frustrated structure with Cr^3+^ cations occupying tetrahedral and octahedral sites, respectively, showing antiferromagnetic behavior at a relatively low Néel temperature of 12.5 K due to its frustrated geometry.^67^ The CT-type phase may also have a magnetically frustrated structure as mentioned above but a completely different geometry from that of the spinel type. This geometry change can make a novel magnetic property, for example, long-range antiferromagnetic ordering as reported in other postspinel phases as mentioned above. Although the present study focused on the crystal chemistry of the CT-type phase and its stability under high pressure and temperature, a further study on measuring the magnetic properties of CT-type ZnCr_2_O_4_ is expected to open a new window for magnetochemistry.

Previous studies intensively investigated the physical properties of CF-type phases such as magnetic and electronic properties and found their specific properties, for example, a complex magnetic ordering with magnetic cycloidal modulation in CaCr_2_O_4_ and nanohalf metallic behavior in NaV_2_O_4_.^5,60,68^ The CT-type structure is close to the CF-type one, but a framework of the former consists of more regular octahedra than those of the latter.^3^ Physical property measurement of the CT-type phase has not yet been performed. It is expected that such an arrangement of transition-metal *B* ^3+^ cations induces magnetic and electric properties that are different from those of CF-type phases. Therefore, it is promising that novel compounds with unique physical properties can be discovered among CT-type structured phases. As demonstrated in this study, the appearance of a CT-type phase can be predicted based on sizes of ionic radii selected. This feature will allow for the formation of a complete solid solution between CT-type compounds with similar-sized *A* cations. Such a solid solution may also have unique properties. In addition, a new postspinel phase can be designed by adding certain amounts of different-sized cations and adjusting ionic sizes of *A*- and *B*-site cations. Thus, in addition to the exploration of new postspinel phases in simple *AB*_2_O_4_ compositions, their exploration in multicomponent systems can further provide new ideas on postspinel crystal chemistry and the physical properties of such phases.

## Conclusions

We have determined high-pressure–temperature phase relations in the ZnCr_2_O_4_ system up to 26 GPa and 1400 °C. We found that the spinel-type phase directly transforms to the CT-type phase with negative temperature dependence at 23–24 GPa and 1000–1400 °C. Thus, our structural categorization for *A* ^*2+*^ *B*_2_^*3+*^O_4_ postspinel phases successfully predicted the structure type of a postspinel phase stabilized in the ZnCr_2_O_4_ system. The Rietveld analysis of the CT-type phase clarified that the A-site octahedral tunnel size in the CT-type structure is adjusted according to the ionic ratio of the A-site cation. This structural feature related to the ionic radius supports that our ionic-radius-based prediction diagram is useful for designing a novel postspinel phase. The present findings will facilitate studies on postspinel phases in the fields of materials science and geoscience.
